# Evaluation of Early Maladaptive Schemas and Adult Attachment Profiles in Patients Diagnosed with Major Depressive Disorder and Examination of Their Relationship with Disease Variables

**DOI:** 10.3390/jcm14010170

**Published:** 2024-12-31

**Authors:** Emre Özaslan, Seda Türkili, Şenel Acar

**Affiliations:** 1 Hatay Reyhanlı State Hospital, 31500 Hatay, Turkey; 2 Faculty of Medicine, Mersin University, 33343 Mersin, Turkey

**Keywords:** attachment, depression, early maladaptive schemas, suicide

## Abstract

**Background/Objectives:** The study aimed to compare the early maladaptive schemas and adult attachment profiles of patients diagnosed with major depressive disorder with those of healthy controls. Another objective of our study was to investigate the potential relationships between disease-related variables—such as the type of depression, number of depressive episodes, history of hospitalization, and suicidal ideation or attempts—and schema and attachment characteristics in the group of patients with major depressive disorder. **Methods:** The study included 118 patients who presented to the Psychiatry outpatient clinic at Mersin University Faculty of Medicine Hospital between 1 April 2021 and 1 September 2021 and were diagnosed with major depressive disorder according to DSM-5 diagnostic criteria based on mental state examinations conducted by researchers, as well as 92 healthy volunteers with no history of mental disorders. A sociodemographic data form prepared by the researchers was used to inquire about characteristics such as gender, age, and educational status. Additionally, a clinical data form was designed and implemented by the researchers to gather information regarding DSM-5 specifiers and the patients’ clinical histories. The Young Schema Questionnaire Short Form-3 was used to evaluate early maladaptive schemas, while adult attachment profiles were assessed using the Experiences in Close Relationships Inventory II. The severity of depression in the patient group was measured with the Beck Depression Inventory. Statistical analysis of the data was conducted using SPSS 21, with a *p*-value less than 0.05 considered statistically significant. **Results:** The study included 118 patients, 84 (71.2%) of whom were women and 34 (28.8%) men, along with 92 healthy volunteers, 60 (65.2%) of whom were women and 32 (34.8%) men. The mean age was 41.9 (±13.2) in the patient group and 40.8 (±11.9) in the control group (*p* > 0.05). The patient group had higher scores than the control group across all schema subtypes and attachment dimensions. Significant differences in certain schemas were observed between patients with chronic depression and those with recurrent depressive episodes, as well as between patients with a single hospitalization history and those with multiple hospitalizations, and between patients with a history of suicide attempts and those without any suicidal ideation or attempts. Positive significant correlations were found between the attachment and schema scores and the severity of depression in both patients and controls. **Conclusions:** Further research is needed to determine the role of schemas and attachment styles in the development of depression in more detail and to focus on schema and attachment-based therapies in treatment.

## 1. Introduction and Objective

Major depressive disorder (MDD) is a significant public health issue affecting millions of people and leading to functional impairments all over the world [1]. In addition to an increased risk of suicide, it causes severe disruptions in individual, familial, social, and economic functioning [2,3,4,5,6]. Steps aimed at reducing the global burden of depression are of great importance. Identifying risk factors across biological, social, and psychological dimensions is crucial for developing effective prevention and intervention strategies.

Interpersonal relationships are known to play a role in the etiology of MDD, alongside various biological, genetic, and psychosocial factors [7]. Early maladaptive schemas are thought to form as a result of the interaction between a child’s temperament and unmet emotional needs during early life [8]. Schemas are cognitive structures acquired in early relationships and effectively regulate emotions, thoughts, and behaviors. Dysfunctional schemas that fail to meet core needs are referred to as early maladaptive schemas [9]. Cognitive theories of depression propose that negative evaluations of life events by individuals constitute a primary risk factor for depression [10,11]. Accordingly, depressive individuals perceive themselves, others, and the world through a negatively biased schematic lens [12].

According to attachment theory, the emotional tone of relationships with caregivers is critical in determining the nature of future close relationships [13]. Attachment is defined as the emotional bond formed with caregivers starting from infancy, stemming from the need for protection. Studies suggest that various attachment styles are more frequently observed in patients with depression [14,15] and that certain attachment styles may be associated with suicide risk [16,17]. In addition, there are studies that various schema subtypes may be more common in depressed patients [18,19], some schemas may be associated with the risk of suicide attempt [20], and schemas may be associated with depression severity and chronicization of depression [21].

Young and colleagues have identified 14 early maladaptive schemas [22], and Ainsworth and colleagues have defined various attachment styles, including secure and insecure types [13]. In this context, studies highlight the relationship between attachment, early maladaptive schemas, and negative effect.

In our study, we aimed to identify the differences in schemas and attachment measurements between MDD patients and a control group with no prior psychiatric diagnoses at a university hospital. Additionally, we sought to investigate the possible relationships between clinical features—such as the type of depression, number of depressive episodes, history of hospitalization, and presence of suicidal ideation or attempts—and schema and attachment characteristics in the patient group. Finally, we aimed to determine whether correlations exist between schema and attachment measurements. We hope that the findings we obtain can be considered as factors contributing to significant outcomes in patients with major depressive disorder, such as the chronicity of the illness, suicide attempts, and the need for hospitalization. In light of these data, we aim to provide insights for the development of prevention and intervention strategies and to address attachment and schema profiles that may serve as targets for attachment-based and schema-focused interventions.

## 2. Methods

### 2.1. Participants and Ethical Approval

This study was conducted with 118 patients diagnosed with MDD according to DSM-5 diagnostic criteria who presented to the Psychiatry Outpatient Clinic of Mersin University Faculty of Medicine Hospital between 1 April 2021, and 1 September 2021, and 92 healthy volunteers (control group) with no psychiatric disorders. Inclusion criteria for the patient group were being aged between 18 and 65 years, literate, having a diagnosis of major depressive disorder based on DSM-5 diagnostic criteria, and signing an informed consent form. Individuals under 18 or over 65 years of age; illiterate; and diagnosed with cognitive impairments such as dementia or intellectual disability, bipolar depressive disorder, depression with psychotic features, psychotic disorders, or alcohol and/or substance use disorders were excluded. The healthy control group included individuals of similar age and gender to the patient group, literate, with no history or current diagnosis of major depressive disorder or other mental disorders, who signed an informed consent form. All participants were informed about the study, and written consent was obtained. Ethical approval for the study was granted by the Clinical Research Ethics Committee of Mersin University (decision dated 31 March 2021, no. 2021/274).

### 2.2. Data Collection Tools

- Sociodemographic and clinical data form: A sociodemographic data form, prepared by the researchers based on similar studies in the literature, assessed characteristics such as age, gender, marital status, and employment status for all participants. A clinical data form was applied to the patient group to evaluate variables such as the type of depression, presence of anxiety symptoms, suicidal ideation or attempts, the number of depressive episodes, and history of inpatient treatment. Patients diagnosed with MDD were questioned about the duration of symptoms, prior depressive episodes, and total number of episodes experienced. Based on DSM-5 criteria, “recurrent depressive episodes” were identified by asking whether there was a full remission period of at least two months after a depressive period and whether similar depressive episodes were experienced before or after this period, along with their number. Similarly, patients without a full remission period of at least two months were assessed for the presence of “chronic depression” by questioning whether symptoms persisted for two or more years, affecting daily professional and social functioning negatively. Participants were also asked whether they had been hospitalized due to depressive disorder and, if so, the number of hospitalizations. The presence and number of suicidal thoughts or attempts during depressive episodes were recorded. To detect accompanying anxiety symptoms, the DSM-5 specifier for “anxious distress” was used. Participants were asked whether they experienced at least two of the following symptoms on most days during depressive episodes: (1) feeling keyed up or tense, (2) unusual restlessness and inability to relax, (3) difficulty concentrating due to worry, (4) fear that something awful may happen, (5) a feeling of losing control. Patients reporting two or more of these symptoms were categorized as having anxious distress.
- Experiences in Close Relationships Inventory II (ECRI-II): Developed by Fraley et al. [23], this 7-point Likert-type scale evaluates anxious and avoidant attachment subdimensions with 18 items each. The Turkish validity and reliability study was conducted by Selçuk et al. [24], with Cronbach’s alpha coefficients for the avoidance and anxiety subscales found to be 0.90 and 0.86, respectively. In our study, these values were determined as 0.84 and 0.78, respectively.
- Young Schema Questionnaire Short-Form III (YSQ-S3): Developed by Jeffrey Young [25], this 90-item, 6-point Likert-type scale measures 14 schema dimensions across five schema domains. The Turkish validity and reliability study was conducted by Soygüt et al. [26], who found the scale reliable and valid for 14 schema dimensions. Cronbach’s alpha coefficients for schema domains ranged between 0.53 and 0.81. In our study, these values ranged from 0.59 to 0.79. Higher scores indicate greater prominence of the respective schema characteristics. The schema dimensions include pessimism, emotional deprivation, failure, social isolation, approval seeking, suppression of emotions, inadequate self-control, interdependence–dependence, imperfection, vulnerability, punishment, high standards, abandonment, and self-sacrifice.
- Beck Depression Inventory (BDI): Developed by Aaron Beck to assess the severity of depression in MDD patients [27], this self-report scale consists of 21 items scored on a 4-point Likert scale, with total scores ranging from 0 to 63. Higher scores indicate more severe levels of depression. The Turkish validity and reliability study was conducted by Hisli [28], with a Cronbach’s alpha coefficient of 0.80. In our study, the Cronbach’s alpha value was found to be 0.79.

### 2.3. Procedure

Between 1 April 2021 and 1 September 2021, patients who presented to the Psychiatry Outpatient Clinic of Mersin University Faculty of Medicine Hospital, met the inclusion and exclusion criteria, and were diagnosed with MDD according to DSM-5 diagnostic criteria based on psychiatric evaluations conducted by the researchers, were informed about the study. Written and verbal consent for voluntary participation was obtained from these patients. Participants with similar age and gender to the patient group, who did not meet DSM-5 criteria for any mental disorder based on mental status evaluation, and who provided verbal and written consent to participate in the study were included in the control group. All participants completed the sociodemographic and clinical data forms prepared by the researchers. Self-report scales were also administered to all participants.

### 2.4. Statistical Analysis

The normality of data distribution was tested using the Shapiro–Wilk test. Continuous variables with normal distribution were summarized with mean and standard deviation, whereas non-normally distributed variables were summarized with median, first quartile (Q1), and third quartile (Q3) values. Descriptive statistics for categorical variables included frequency (*n*) and percentage (%).

Comparisons of means between two independent groups were conducted using the Student’s *t*-test, a parametric test. Comparisons of medians between two independent groups were performed using the Mann–Whitney U test, a non-parametric test. For comparisons of medians among more than two independent groups, the Kruskal–Wallis test, a non-parametric test, was utilized. Post hoc analyses to determine which groups contributed to statistically significant differences in the Kruskal–Wallis test were conducted using the Bonferroni-corrected post hoc test.

Relationships between categorical variables were analyzed using Chi-square tests, and the relationship between continuous variables was analyzed using correlation analysis. Correlation coefficients were interpreted as follows: “correlation coefficient = 0.00–0.49: weak; 0.50–0.69: moderate; 0.70–0.89: high; 0.90–1.00: very high”. Statistical analysis of the data was conducted using SPSS (Statistical Package for the Social Sciences; SPSS Inc., Chicago, IL, USA) version 21. For all comparisons, a *p*-value of ≤0.05 was considered statistically significant.

### 2.5. Findings

Our study included 118 patients diagnosed with MDD according to DSM-5 criteria and 92 healthy volunteers with no psychiatric diagnosis. The mean age of the patient group was 41.97 ± 13.28, while the control group’s mean age was 40.86 ± 11.94. No significant differences were found between the groups in terms of age, gender, or marital status. Other sociodemographic data of the participants are presented in Table 1.

In the major depressive disorder group, the proportion of individuals actively engaged in income-generating employment was found to be lower compared to healthy controls, while the rates of being a housewife or unemployed were higher. Regarding participants’ place of residence, it was observed that the patient group predominantly resided in urban centers, whereas the proportion of individuals living in rural areas was higher in the control group.

In the MDD group, disease-specific clinical variables were assessed, including the type of depression (first episode, recurrent episodes, or chronic depression), the presence of accompanying anxiety symptoms, the number of depressive episodes, the presence of suicidal thoughts and attempts, and the number of hospitalizations. The relevant findings are summarized in Table 2.

When comparing the attachment, schema, and BDI scores of the patient and control groups, the ECRI-II avoidance and anxiety scores, all schema subscale scores, and BDI scores were found to be significantly higher in the patient group compared to the control group. The relevant variables are presented in Table 3.

In the patient group, no significant differences were observed in attachment scores between those who received inpatient treatment and those who did not. However, the median scores for the intertwining-dependency and defectiveness/shame schema subscales were significantly higher in the inpatient group (*p*-values = 0.023 and 0.035, respectively). Among those who had received inpatient treatment, no significant differences in attachment scores were found between patients with multiple hospitalization histories and those with only one hospitalization. However, significant differences were identified in the median scores for the failure, privilege/insufficient self-control, and approval-seeking schema subscales (*p*-values = 0.011, 0.032, and 0.027, respectively). The median pessimism score was significantly higher in patients with accompanying anxiety symptoms compared to those without (*p* = 0.015).

Patients with chronic depression had higher ECRI-II anxiety scores than those with recurrent depression (*p* < 0.05). Likewise, BDI scores were significantly higher in patients with chronic depression compared to those experiencing their first depressive episode (*p* = 0.013) and those with recurrent depression (*p* = 0.005). Patients with chronic depression also had significantly higher scores on the defectiveness/shame (*p* = 0.014), abandonment (*p* = 0.044), and failure (*p* = 0.007) schema subscales compared to those with recurrent depression.

Patients with a history of suicide attempts scored significantly higher on the emotional deprivation, social isolation, abandonment, and defectiveness/shame schema subscales compared to those with no history of suicidal ideation or attempts (*p* < 0.05). However, there were no significant differences in attachment scores.

Correlations between attachment styles, schema subscales, and BDI scores for the patient and control groups are presented in Table 4. Accordingly, a moderate positive correlation was found between ECRI-II avoidance and emotional inhibition (suppression of emotions) in both the patient and control groups. In the patient group, a moderate positive correlation was identified between anxious attachment and both social isolation and abandonment. In the control group, moderate positive correlations were observed between pessimism and abandonment. Additionally, in both groups, all schema scores except approval-seeking demonstrated weak but significant positive correlations with BDI scores.

## 3. Discussion

In our study, anxious and avoidant attachment scores were significantly higher in patients compared to the control group. This finding is in line with publications indicating that anxious and avoidant attachment, which are insecure attachment styles, are higher in MDD patients compared to the general population [15,29,30]. In a study conducted with university students in China, it was stated that participants with anxious and avoidant types of insecure attachment had more depressive and anxious symptoms at 6-month follow-up [31].

In our study, the median value for anxious attachment in the patient group was statistically significantly higher in the group with chronic depression than in the group with recurrent depression. In a seven-year prospective follow-up study, it was reported that depressive patients with both anxious and avoidant attachment had shorter symptom-free periods, and insecure attachment increased the risk of relapse and was associated with unfavorable prognosis [32]. There is also a study reporting that both types of insecure attachment are risk factors for the chronicization of depression [33].

In our patient group, we did not find a significant difference in attachment scores between those who had suicidal thoughts or attempts and those who had no suicidal thoughts or attempts. Similar to our finding, in the study conducted by Özer et al. [15] in depressive patients, no significant difference was found between both anxious and avoidant attachment scores of depressive patients with and without suicidal thoughts, but unlike our study, when they compared the study design with the control group, they found a higher rate of suicide attempts in insecurely attached patients compared to the control group. Studies show that insecure attachment style, mostly anxious attachment, is associated with increased suicide risk [34]. There is a study that explains the high rate of suicidal ideation/behavior in anxious attachment in the context of interpersonal relationships, lack of sociability in people with this attachment style, being more sensitive, and social isolation, and suggests that social isolation mediates [35]. This finding is consistent with the finding that the social isolation schema score was higher in our group of patients with suicide attempts compared to the group without suicide attempts.

When we looked at the correlation of anxious or avoidant attachment scores with BDI scores, we found a positive, weakly significant correlation between attachment score and BDI score in the patient group for both anxious and avoidant attachment. There is a study reporting a correlation between depression severity and insecure attachment in depressive patients admitted to primary care [36]. In a sample of approximately seven hundred people who applied for psychiatric treatment, anxious and avoidant attachment styles showed a moderate positive correlation with depression severity in those who reported being exposed to interpersonal trauma [37]. Although we do not have information about other possible factors affecting the severity of depression in our study, the higher correlation between insecure attachment scores and depression severity in our patient group compared to the control group may raise the possibility of the contribution of insecure attachment or other related factors to the stress diathesis model in the etiology of depression.

When the participants were evaluated for early maladaptive schemas, we found that the scores for all 14 schemas were significantly higher in our patients than in the control group. In terms of the five main schema domains, similar to our study, there is a study [20] that found the overall schema scores high in depressive patients, as well as studies that found some sub-schema scores high. By comparing depressive patients with healthy controls, it was found that depressive patients were higher in schemas such as failure, social isolation, pessimism, abandonment, defectiveness, and vulnerability to harm [38] and in thirteen other schema domains except for the justification–aggrandizement schema [39], some of the studies that found high scores in most schema areas such as insecurity, abandonment, defectiveness, social isolation, emotional deprivation, resilience, failure, addiction, approval seeking, submissiveness, and altruism [40]. In a 2021 meta-analysis, fourteen schema dimensions were reported to be associated with depression [41].

We found that the schema values for defectiveness, abandonment, and failure were higher in individuals with chronic depression compared to those with recurrent depression. In a study comparing the five core schema domains between patients with chronic depression and those with single-episode depression, higher schema scores were observed in patients with persistent depression for the domains of rejection, separation, over-vigilance/inhibition, and impaired autonomy [21]. Furthermore, the same study identified separation–rejection and hyper-vigilance/abstinence as the two best predictors of depression severity. Notably, two schema subtypes (defectiveness and abandonment), which were found to be elevated in our study, also belong to these domains.

In a nine-year longitudinal follow-up study, a relationship was observed between the schema domain of impaired autonomy and the development of depression in individuals without prior depressive episodes [39]. The failure schema, which showed elevated scores in our study, also belongs to this domain.

Risk factors for depression, such as parental loss, neuroticism (a trait characterized by sensitivity and a tendency to feel guilt), and instability in relationships with significant others (as described in the abandonment/instability schema), highlight overlapping characteristics with these schemas. In this context, it can be hypothesized that patients with high scores across all schemas may have an increased likelihood of chronicity in their depression due to their elevated schema scores in these domains.

In our patient group, we observed weak yet statistically significant positive correlations between Beck Depression Inventory (BDI) scores and all schemas except for the approval-seeking schema. In a general sample, a study by Cormier et al. [19] reported a correlation between schema scores and the severity of depression among depressed participants. Additionally, specific schemas, such as abandonment, emotional deprivation, and failure, have been identified as being associated with the severity of depressive symptoms in other studies [42]. These findings highlight the relationship between early maladaptive schemas and the intensity of depressive symptoms, supporting the idea that certain schemas may play a more prominent role in influencing the severity of depression.

In our patient group, we observed significantly higher scores in four schemas (emotional deprivation, social isolation, abandonment, and failure), all of which belong to the separation–rejection schema domain, among individuals with a history of suicide attempts compared to those without any suicidal thoughts or attempts. Similarly, a study by Ahmadpanah et al. [20] reported higher scores in the same four schema subtypes in major depressive disorder (MDD) patients with suicide attempts compared to both non-suicidal depressed patients and healthy controls. Furthermore, another study investigating the potential relationship between early maladaptive schemas, schema modes, and suicide risk found that the separation–rejection schema domain, one of the five core schema domains, was significantly associated with suicide risk [43].

These findings suggest that schemas within the separation–rejection domain play a crucial role in suicide risk among individuals with depression, highlighting their importance in both assessment and therapeutic interventions aimed at reducing suicidality.

In a study comparing a group of suicide attempt patients with depression and anxiety symptoms with controls without suicide attempts, it was stated that emotional deprivation and defectiveness schemas were associated with both negativity in parental attachment style and recurrent suicide risk [44].

The social isolation schema has also been identified as linked to suicidal thoughts and behaviors in our study, as well as in studies involving depressive patients [45,46] and bipolar patients [47]. Additionally, a study by Stepp et al. [35] emphasized the mediating role of social isolation in suicidal thoughts and behaviors among insecurely attached individuals, underscoring its significance as a potential contributing factor to suicidality. These findings collectively highlight the crucial role of the emotional deprivation, defectiveness, and social isolation schemas in understanding suicidal behavior and underscore the importance of targeting these schemas in therapeutic interventions aimed at reducing suicide risk.

In our patient group, we observed significantly higher scores for the intertwining/dependency and defectiveness schemas among inpatients compared to those with no history of hospitalization. This finding may align with the increased need for social support and reliance on others observed in individuals experiencing a depressive mood. Alternatively, these characteristics might contribute to a greater frequency of depressive episodes and a higher likelihood of requiring inpatient treatment.

The elevated intertwining/dependency schema suggests a heightened reliance on close relationships for emotional support, potentially reducing resilience in coping with stressors independently. Similarly, the defectiveness schema, characterized by persistent feelings of inadequacy and self-perceived flaws, may exacerbate depressive symptoms and increase the severity of depressive episodes, ultimately leading to hospitalization. These observations underscore the importance of addressing these specific schemas in therapeutic interventions for individuals with depression, particularly those requiring inpatient care, to enhance their coping mechanisms and reduce dependence on external support systems.

In both the patient and control groups, we identified correlations between several schema subtypes and attachment style scores. For instance, the abandonment schema score showed a moderate, statistically significant positive correlation with anxious attachment scores in both groups. The social isolation schema score demonstrated a moderate positive correlation with anxious attachment in the patient group, while this correlation was weak yet statistically significant in the control group. Other schemas exhibited weak positive correlations with anxious attachment scores in both the patient and control groups.

These findings suggest that attachment styles, particularly anxious attachment, may play a significant role in the development and maintenance of maladaptive schemas, with a stronger association observed in clinical populations. Addressing attachment-related issues in therapeutic interventions may therefore be crucial for effectively targeting maladaptive schemas in individuals with depression.

The emotional inhibition schema score demonstrated a moderate, statistically significant positive correlation with avoidant attachment scores in both the patient and control groups. Additionally, schema scores for emotional deprivation, social isolation, failure, interdependence/dependence, abandonment, vulnerability to threats, and defectiveness exhibited weak yet statistically significant positive correlations with avoidant attachment scores across both groups. In the patient group specifically, schema scores for pessimism, approval-seeking, privilege/insufficient self-control, punitiveness, and high standards also showed weak but statistically significant positive correlations with avoidant attachment scores.

These findings suggest a consistent association between avoidant attachment and various maladaptive schemas, with stronger patterns emerging in clinical populations. The link between emotional inhibition and avoidant attachment is particularly noteworthy, as it reflects a defensive strategy often used to suppress emotional expression in relationships, potentially contributing to the maintenance of depressive symptoms. Addressing attachment dynamics, particularly avoidant attachment tendencies, in schema-focused therapeutic approaches may enhance outcomes for individuals struggling with these maladaptive schemas.

A study investigating the relationship between attachment scores and schema scores using a method that measures the secure base effect, which lies at the core of attachment relationships, found significant associations between both anxious and avoidant attachment styles and schema scores. The same study reported that individuals with higher scores for consistent secure base experiences had lower scores in early maladaptive schemas within the separation–rejection schema domain [48].

In a longitudinal study following participants over 15 years from childhood to adulthood, individuals with insecure attachment during childhood exhibited higher scores across multiple schema domains in adulthood. However, no significant differences were found between different subtypes of insecure attachment in terms of schema scores [49]. Another study indicated that individuals who experienced parental separation during childhood had higher insecure attachment scores in adulthood. Additionally, the abandonment schema was found to mediate the relationship between childhood parental separation and adult anxious and avoidant attachment styles [50]. These findings underscore the complex interplay between early attachment experiences and the development of maladaptive schemas. Secure attachment, particularly through the secure base effect, appears to serve as a protective factor against the development of maladaptive schemas, whereas insecure attachment—regardless of its specific subtype—tends to be associated with higher schema scores and increased vulnerability to psychological distress.

## 4. Conclusions and Recommendations

In the group of patients diagnosed with major depressive disorder (MDD), significantly higher scores were observed in insecure attachment measures and all 14 subtypes of early maladaptive schemas compared to the control group.

Our study contributes to a cross-sectional understanding of the existing schemas and attachment characteristics in patients with depression. Descriptive findings have been obtained regarding schemas and attachment patterns that are more prominent in depressed patients and those potentially influencing chronicity and suicidal thoughts or behaviors. Different schema domains were found to be associated with distinct types or features of depression. Although the cross-sectional design prevents establishing causal relationships between findings and current conditions, our study supports previous research and provides a theoretical foundation for further in-depth investigations. A more comprehensive exploration of the interaction between schemas, attachment patterns, and other etiological factors in depression may facilitate the identification of individuals at risk for the development and chronicity of depression. This, in turn, could enable the implementation of early therapeutic interventions. Additionally, our findings may help clarify the schema and attachment domains that require focused attention in schema- and attachment-based therapies for patients diagnosed with MDD. Future longitudinal studies are recommended to explore these relationships more systematically and uncover causal mechanisms to inform targeted therapeutic approaches.

## 5. Strengths and Limitations of the Study

There are relatively few studies in the literature investigating the relationship between early maladaptive schemas, attachment styles, and major depressive disorder (MDD) in the Turkish population. In this context, our study contributes valuable insights into the role of attachment profiles and maladaptive schemas as potential risk factors for the development of depression.

Incorporating therapeutic approaches targeting attachment styles and maladaptive schemas into the treatment of MDD, along with developing interventions focused on specific schema domains, could enhance treatment efficacy. This approach may reduce adverse outcomes such as relapse, chronicity, hospitalization needs, and suicide attempts.

The study was conducted between 1 April 2021 and 1 September 2021, coinciding with the period of COVID-19 restrictions in Turkey. As a result, access to participants was limited, which may have impacted the sample size. Additionally, the cross-sectional design of the study prevents the establishment of causal relationships between the observed variables, representing another limitation.

Despite these limitations, the findings of this study provide a strong foundation for future longitudinal and experimental research exploring the complex interplay between early maladaptive schemas, attachment styles, and depression.

## Figures and Tables

**Table 1:** Sociodemographic characteristics of all participants.

Sociodemographic Variables	Patient Group *n* (%)	Control Group *n* (%)	*p*
Gender			0.355
Male	34 (28.8)	32 (34.8)	
Female	84 (71.2)	60 (65.2)	
Marital status			0.062
Married	73 (61.9)	69 (75)	
Single	33 (28)	20 (21.7)	
Divorced	12 (10.2)	3 (3.3)	
Employment status			**<0.0001**
Actively working	48 (40.7)	64 (69.6)	
Student	12 (10.2)	9 (9.8)	
Housewife	31 (26.3)	11 (12)	
Retired	13 (11)	8 (8.7)	
Unemployed	14 (11.9)	0 (0)	
Place of residence			**0.031**
Rural	22 (18.6)	29 (31.5)	
Urban center	96 (81.4)	63 (68.5)	
Family history of psychiatric illness			**<0.0001**
None	62 (52.5)	84 (91.3)	
MDD	42 (35.6)	2 (2.2)	
Other	14 (11.9)	6 (6.5)	
Substance use			**0.043**
None	65 (55.1)	57 (62.0)	
Cigarette	33 (28.0)	15 (16.3)	
Alcohol	4 (3.4)	10 (10.9)	
Cigarette + alcohol	16 (13.6)	10 (10.9)	

*p*-values in bold: Values of *p* < 0.05 are considered statistically significant. MDD: major depressive disorder.

**Table 2:** Disease-specific variables in the patient group.

Disease-Specific Variables	*n* (%)	Disease-Specific Variables	*n* (%)
Form of depression		Number of attacks	
First episode	40 (33.9)	Single attack	40 (38.8)
Recurrent episodes	64 (54.2)	Two attacks	39 (37.9)
Chronic depression	14 (11.9)	Three or more attacks	24 (23.3)
Concomitant anxiety symptoms		Inpatient treatment	
No	27 (22.9)	No	95 (80.5)
Yes	91 (77.1)	Yes	23 (19.5)
Suicidal thoughts/attempts		Number of hospitalizations (*n* = 23)	
None	58 (49.2)	One hospitalization	12 (52.2)
Only thoughts	33 (28.0)	Two or more hospitalizations	11 (47.8)
Attempted	27 (22.9)		

**Table 3:** Median/mean values of attachment, schema, and BDI of patient and control group.

	Patient (*n* = 118)			Control (*n* = 92)			
	Median	Q1	Q3	Median	Q1	Q3	*p*
ECRI Avoidance	63.5000	48.7500	76.2500	46.5000	35.0000	61.7500	<0.0001 *
Emotional deprivation	14.5000	9.0000	21.0000	7.5000	5.0000	10.7500	<0.0001 *
Failure	14.0000	9.0000	20.2500	10.0000	7.0000	12.7500	<0.0001 *
Pessimism	17.0000	11.0000	22.0000	9.0000	6.0000	14.0000	<0.0001 *
Social isolation	24.0000	18.0000	29.2500	13.5000	11.0000	17.0000	<0.0001 *
Suppressing emotions	14.0000	9.7500	19.0000	9.0000	6.0000	13.0000	<0.0001 *
Nesting	21.5000	15.0000	30.0000	9.0000	6.0000	13.0000	<0.0001 *
Self-sacrifice	21.0000	15.0000	25.0000	16.0000	11.0000	21.0000	<0.0001 *
Abandonment	10.5000	7.0000	16.0000	7.0000	5.0000	10.0000	<0.0001 *
Punishment	25.0000	20.0000	29.0000	19.0000	16.0000	26.0000	<0.0001 *
Imperfection	12.5000	8.0000	18.2500	7.0000	6.0000	10.0000	<0.0001 *
Vulnerability to threats	14.0000	11.0000	19.0000	10.0000	8.0000	14.0000	<0.0001 *
High standards	11.0000	7.0000	14.0000	8.0000	5.0000	12.0000	<0.0001 *
BDE	23.00	14.00	32.25	9.00	6.00	14.00	<0.0001 *
	**Patient (*n* = 118)**			**Control (*n* = 92)**			
	**Mean**	**±**	**St.dv**	**Mean**	**±**	**St.dv**	***p***
ECRI anxiety	69.4831	±	17.98039	54.5652	±	18.654	<0.0001 ^¶^
Seeking approval	22.8729	±	6.57052	18.7717	±	6.3108	<0.0001 ^¶^
Privilege insufficient self-regulation	25.6949	±	7.54531	22.2717	±	7.291	0.001 ^¶^

*: Mann–Whitney U test; ^¶^: Student’s *t*-test.

**Table 4:** Attachment–schema–BDI correlation analysis of the control group and patient group.

	Control Group *n* = 92			Patient Group *n* = 118	
	ECRI Anxiety Score		ECRI Avoidance Score	ECRI Anxiety Score	ECRI Avoidance Score
Emotional deprivation	r	0.419	0.470	0.352	0.295
	*p*	0.00 *	0.00 *	0.00 *	0.001 *
Failure	r	0.439	0.244	0.364	0.274
	*p*	0.00 *	0.019 *	0.00 *	0.003 *
Pessimism	r	0.527	0.186	0.469	0.302
	*p*	0.00 **	0.075	0.00 *	0.001 *
Social isolation insecurity	r	0.471	0.343	0.513	0.300
	*p*	0.00 *	0.001 *	0.00 **	0.001 *
Suppressing emotions	r	0.429	0.501	0.325	0.544
	*p*	0.00 *	0.00 **	0.00 *	0.00 **
Seeking approval	r	0.457	−0.034	0.484	0.274
	*p*	0.00 *	0.748	0.00 *	0.003 *
Nesting dependency	r	0.457	0.424	0.408	0.303
	*p*	0.00 *	0.00 *	0.00 *	0.001 *
Privilege insufficient self-control	r	0.257	0.133	0.325	0.208
	*p*	0.014 *	0.206	0.00 *	0.024 *
Self-sacrifice	r	0.351	0.118	0.221	0.075
	*p*	0.001 *	0.263	0.016 *	0.421
Abandonment	r	0.578	0.308	0.573	0.284
	*p*	0.00 **	0.003 *	0.00 **	0.002 *
Punishment	r	0.268	0.001	0.302	0.251
	*p*	0.01 *	0.996	0.001 *	0.006 *
Imperfection	r	0.391	0.388	0.446	0.390
	*p*	0.00 *	0.00 *	0.00 *	0.00 *
Lack of resilience in the face of threats	r	0.480	0.260	0.428	0.338
	*p*	0.00 *	0.012 *	0.00 *	0.00 *
High standards	r	0.473	0.137	0.226	0.218
	*p*	0.00 *	0.191	0.014 *	0.018 *
BDI score	r	0.269	0.273	0.365	0.350
	*p*	0.01 *	0.008 *	0.00 *	0.00 *

r: correlation coefficient; *p*: *p*-value for statistical significance of correlation coefficient. *: weak; **: moderate strong correlation. ECRI: Experiences in Close Relationships Inventory; BDI: Beck Depression Inventory.

## Data Availability

The original contributions presented in this study are included in the article. Further inquiries can be directed to the corresponding author.
